# Mimicking normal tissue architecture and perturbation in cancer with engineered micro-epidermis

**DOI:** 10.1016/j.biomaterials.2012.04.009

**Published:** 2012-07

**Authors:** Julien E. Gautrot, Chunming Wang, Xin Liu, Stephen J. Goldie, Britta Trappmann, Wilhelm T.S. Huck, Fiona M. Watt

**Affiliations:** a Melville Laboratory for Polymer Synthesis, Department of Chemistry, University of Cambridge, Lensfield Road, Cambridge, CB2 1EW, UK; b Wellcome Trust Centre for Stem Cell Research, University of Cambridge, Tennis Court Road, Cambridge, CB2 1RE, UK; c School of Engineering and Materials Science, Queen Mary, University of London, Mile End Road, London, E1 4NS, UK; d Cancer Research UK Cambridge Research Institute, Li Ka Shing Centre, Robinson Way, Cambridge, CB2 0RE, UK; e Radboud University Nijmegen, Institute for Molecules and Materials, Heyendaalseweg 135, 6525 AJ Nijmegen, The Netherlands

**Keywords:** Polymer brush, Micro-patterning, Keratinocyte, Morphogenesis, Cadherins, Cancer

## Abstract

Correct tissue architecture is essential for normal physiology, yet there have been few attempts to recreate tissues using micro-patterning. We have used polymer brush micro-engineering to generate a stratified micro-epidermis with fewer than 10 human keratinocytes. Epidermal stem cells are captured on 100 μm diameter circular collagen-coated disks. Within 24 h they assemble a stratified micro-tissue, in which differentiated cells have a central suprabasal location. For rings with a non-adhesive centre of up to 40 μm diameter, cell–cell and cell–matrix adhesive interactions together result in correct micro-epidermis assembly. Assembly requires actin polymerization, adherens junctions and desmosomes, but not myosin II-mediated contractility nor coordinated cell movement. Squamous cell carcinoma cells on micro-patterned rings exhibit disturbed architecture that correlates with the characteristics of the original tumours. The micro-epidermis we have generated provides a new platform for screening drugs that modulate tissue assembly, quantifying tissue stratification and investigating the properties of tumour cells.

## 1 Introduction

Recent advances in micro-patterning technology have made it possible to identify key microenvironmental cues that regulate stem cell behaviour at single cell resolution [1,2]. However, the architectural and functional complexity of tissues is essential for their physiology [3,4], yet there have been few attempts to use micro-patterning to recreate tissues *in vitro* [5,6]. This is an important goal because it opens the way for designing screens for small molecules that modulate tissue physiology and a platform for uncovering disease mechanisms that operate at the level of groups of cells rather than at the single cell level.

**Fig. 1:**
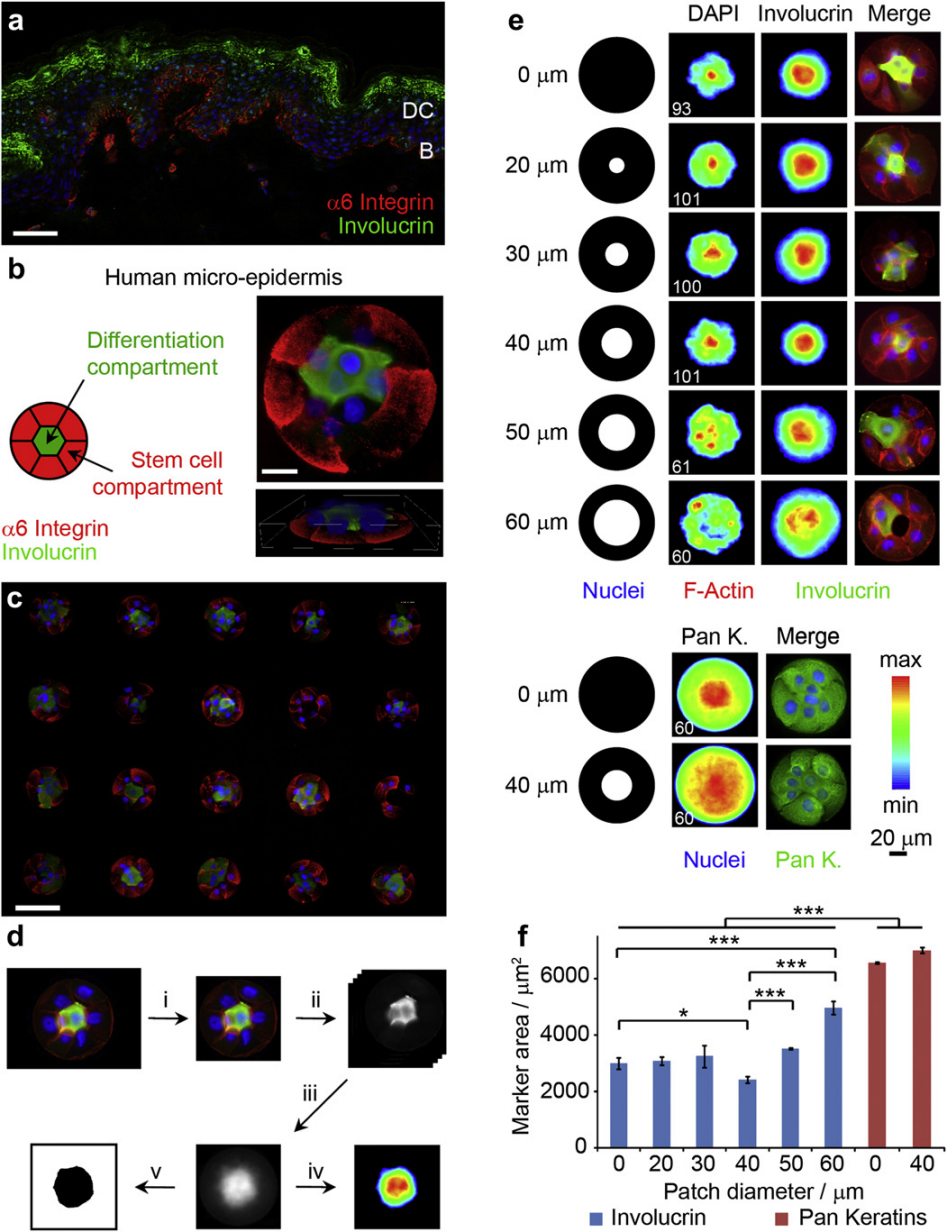
Formation of micro-epidermis mimicking some of the features of normal tissue. (a) human skin section immuno-stained for involucrin (green), α6 integrin (red) and DAPI (blue). Scale bar 50 μm. DC, differentiation compartment; B, basal stem cell compartment. (b) Right, example of single cluster 2D (top, scale bar 20 μm) and 3D (bottom, box width 110 μm, height 15 μm) images. Left, schematic representation of compartmented micro-epidermis. (c) Micro-epidermis array immuno-stained for involucrin (green), α6 integrin (red) and DAPI (blue). Scale bar 100 μm. (d) Protocol used for image analysis: i, images were cropped; ii, channels were stacked and split; iii, stacks were overlayed; iv, heatmaps were generated with 30% intensity threshold; v, image thresholding (30% intensity) allowed the determination of marker areas. (e) Undifferentiated keratinocytes adhered to collagen I disks and rings for 24 h before fixation and immuno-staining. Adhesive island geometries (in black) for collagen rings with protein resistant patches ranging from 0 to 60 μm in diameter. Heatmaps (left and middle columns) and single cluster images (right column; involucrin and for the two bottom rows pan keratins, green; F-Actin, Red; DAPI, blue). White numbers (bottom left of heatmaps) are the number of images overlayed. (f) Involucrin (blue) and pan keratins (red) areas extracted from heatmaps. *P < 0.1; ***P < 0.01; n.s. non significant, *P* > 0.1; error bars are s.e.m., *n* ≥ 3(For interpretation of the references to colour in this figure legend, the reader is referred to the web version of this article.).

Human epidermis is an obvious tissue to engineer at the micro-scale. The interfollicular epidermis is a multi-layered epithelium in which the basal layer of cells is attached to an underlying extracellular matrix (ECM), known as the basement membrane, and the suprabasal layers comprise cells that undergo terminal differentiation, culminating in formation of the barrier that protects the body from water loss and penetration by micro-organisms. As the outermost cells are shed from the surface of the epidermis they are replaced by proliferation of stem cells in the basal layer. There are well-characterised markers of the terminal differentiation process, including involucrin and transglutaminase 1 (Fig. 1a) and a number of markers that enrich for stem cells, including β1 integrins, Lrig1 and Dll1. Cultured epidermis is used to provide long-term autologous grafts for burns victims, providing evidence that stem cells persist in culture. In addition, cells can be cultured from tumours of the epidermis and other multi-layered epithelia, such as the oral cavity, and these can be used to study changes in cell behaviour linked to cancer.

We, and others, have previously used micro-patterning techniques to culture single cells on ECM-coated islands of defined shape and size [1,2,7,8]. Here, we used micro-patterned substrates for quantitative analysis of the architecture of multi-cellular structures. This allowed us to compare the differentiation and assembly of epidermal stem cells and cells derived from squamous cell carcinomas (SCCs) in bi-compartment structures that mimic or deviate from the architecture of normal epidermis.

## 2 Materials and methods

### 2.1 Preparation of micro-patterned substrates

Micro-patterned slides suitable for cell culture were prepared by first depositing a monolayer of ω-mercaptoundecylbromoisobutyrate (5 mM ethanolic solution) on a thin gold-coated glass slide (1.5 nm chromium, 15 nm gold, Edwards Auto 500 evaporator, thickness 1 borosilicate glass) using a PDMS stamp (184 silicone elastomer, Sylgard) [7]. The stamp was generated by casting against a SU8-2005 photoresist (MicroChem) master (spin-coated on Si wafer to a final film thickness of 5 μm exposed to UV light through a photomask from Micro Lithography Services Ltd). To generate the polymer brush (poly oligo(ethylene glycol methyl ether methacrylate), POEGMA) coating, the resulting micro-patterned slides were then placed in a degassed (nitrogen bubbling) solution of CuBr_2_ (9 mg, 40 mmol), CuCl (41 mg, 410 mmol), bpy (160 mg, 1.0 mmol) and OEGMA (6.3 g, 17.5 mmol) in water/ethanol 4/1 (15 mL) for 15 min under inert atmosphere (nitrogen). The polymer brush patterned slides were washed with copious amounts of water and ethanol and stored in ambient conditions until needed for cell patterning.

For collagen I (BD Biosciences) deposition, cut micro-patterned substrates (0.8 × 0.8 cm squares) were placed in a 24-well plate, sterilized with 70% ethanol for 5 min, washed twice with PBS and incubated in a collagen I solution (0.5 mL, 20 μg/mL in PBS) at room temperature for 60 min. Substrates were washed by first diluting with PBS and aspirating the resulting solution without allowing the substrates to dry and repeating this dilution/aspiration protocol twice. Finally, substrates were washed twice with PBS and used shortly after for cell seeding.

### 2.2 Culture of primary human keratinocytes and cell seeding

Primary human epidermal keratinocytes (HEKs) were isolated from neonatal foreskin and were cultured with feeder cells (J2 3T3 fibroblasts) as previously described (FAD medium used for culture was prepared as follows: 1 part Ham's F12, 3 parts DMEM, 10% FBS, 0.5 mg/ml hydrocortisone, 5 mg/ml insulin, 10^−10^ M cholera toxin, 10 ng/ml EGF) [9]. The feeders were removed using a solution of versene (no trypsin, Gibco) before detachment of HEKs (passage 2–8) using trypsin (0.25% from Gibco, dilution of versene/trypsin 9/1 from stock solutions) and re-seeded onto the micro-patterned substrates (pre-coated with collagen I, in a 24-well plate) at a density of 75,000 cell cm^−2^ (150,000 cells/mL, 0.5 mL/well) in FAD medium. For 50 and 200 μm collagen islands, the cell densities used were 25,000 and 125,000 cell cm^−2^, respectively. Cell adhesion was allowed to proceed for 1 h before rinsing with fresh medium (three times 0.5 mL). Care was taken not to let the substrate dry during this washing step.

HEKs expressing a dominant negative E-cadherin (DNC) construct were prepared by sub-cloning the cDNA into the retroviral vector pBabe puro and subsequent transducing into cultured HEKs with a two step co-culture procedure [10]. The DNC construct encodes a chimeric protein fusing the extracellular part of H-2Kd and the transmembrane and cytoplasmic domains of mouse E-cadherin [11]. An empty vector (EV) was used as negative control. Infected AM12 cells were selected with puromycin (2 μg/ml) for 3 days, de-activated with mitomycin (4 μg/ml, 3 h) and re-seeded at 3.5 × 10^6^ cells per T75 flask. The next day, HEKs (passage 3) were plated at 3.10^5^ × cells per flask onto these AM12 feeders in complete FAD medium. After 3 days of co-culture and infection, the AM12 cells were removed with versene, mitomycin-treated J2 puro feeders were added (3.5 × 10^6^ cells per T75 flask) and selection was carried out with puromycin (2 μg/ml) for 3 days.

For isolation and preparation of cancer cell lines, small biopsy specimens were removed from freshly resected oral SCCs. SJG13 cells were derived from a SCC (pathological stage T4aN0M0; well differentiated) on the upper alveolus of a 54-year old female patient. SJG15 cells were derived from a SCC (pathological stage T2N1M0; well differentiated) on the lateral tongue of a 51-year old female patient. SJG25 cells were derived from a SCC (pathological stage T2N0M0; well differentiated) on the alveolar ridge of a 59-year old male patient. SJG26 cells were derived from a SCC (pathological stage T4aN1M0; moderately differentiated) on the lateral tongue of a 74-year old male patient. Cells isolated from tumours were disaggregated in 0.25% trypsin/versene at 37 °C then grown in complete FAD medium on J2 3T3 feeders until they became feeder-independent.

Work with human material was carried out in compliance with the UK Human Tissue Act (2004) and approved by the National Research Ethics Service (08/H0306/30). Appropriate informed consent was obtained from patients diagnosed with oral SCC, prior to operation at Addenbrooke's Hospital.

### 2.3 Antibodies, inhibitors and siRNA knock down

The rabbit anti-Ki67 (1:1000), anti-keratins (1:250) and mouse anti-P-cadherin (1:100, clone 6A9) antibodies were purchased from Abcam. The mouse anti-vinculin (hVIN1; 1:200), the rabbit anti-myosin IIa (1:200), anti-GAPDH (1:1000), anti-nucleophosmin (1:500) and TRITC-phalloidin (1:500) were from Sigma Aldrich. Rat anti-α6 integrin (1:1000, clone GoH3) was from BD Pharmingen. The mouse anti-β1 integrin (1:200, clone P5D2) was from Developmental Studies Hybridoma Bank. The mouse anti-E-cadherin (1:100, HECD1) was a gift from M. Takeichi (Kyoto University). The mouse anti-desmoplakin (1:100, clone 115F) was a gift from D. R. Garrod. The Mouse anti-involucrin (SY7; 1:1000) and mouse anti-transglutaminase I (BC1; 1:500) were prepared by Cancer Research UK central services.

Blebbistatin and cytochalasin D were obtained from Calbiochem (La Jolla, CA). These inhibitors were introduced in the medium when washing the excess cells, after 1 h incubation.

For siRNA transfection, HEKs were cultured in KSFM on collagen-coated dishes for 24 h before transfection. Cells were transfected in KSFM free of supplements for 3 h using Lipofectamine 2000 (Invitrogen, standard manufacturer protocol) and subsequently cultured in KSFM for two days prior to harvesting and seeding onto micro-patterned substrates. Non-targeting, E-cadherin (CDH1), P-cadherin (CDH3) and desmoplakin (DSP) siRNAs were from Qiagen (target sequences, CDH1, SI02654029, TCGGCCTGAAGTGACTCGTAA, CDH3, SI02663934, CAGATGAAATCGGCA-ACTTTA, DSP, SI00374402, CAGAAGAATGACTATGACCAA). For western blot analysis, cells were re-seeded in collagen I coated 6-well plates at a density of 3 × 10^5^ cells per well in complete FAD, cultured for 24 h and lysed in RIPA buffer supplemented with protease and phosphatase inhibitors.

### 2.4 Immuno-fluorescence and microscopy

For immuno-fluorescence staining, cells were fixed in paraformaldehyde (4%, 10 min), permeabilized with Triton X-100 (0.2%, 5 min) and blocked for 1 h (10% FBS plus 0.25% gelatin) at room temperature. Samples were incubated with primary antibodies for 1 h at room temperature, washed, incubated with conjugated secondary antibodies (1:1000; Alexafluor 488 and 555; Invitrogen) for 1 h and finally washed, at room temperature. TRITC-phalloidin was included in the blocking solution and DAPI (4,6-diamidino-2-phenylindole) was included in the secondary antibody solution, where indicated. Samples were mounted on glass slides with Mowiol reagent (4-88 Reagent, Calbiochem).

Fluorescence microscopy images were acquired with a Leica DMI4000 fluorescence microscope (CTR 6000 laser, excitation filter BP480/40, suppression filter B527/30, dichromatic mirror 505). 3D Fluorescence imaging was carried out using a Zeiss Axio Observer, using an ApoTome (Zeiss) for sectioning (z-stack consisting of 0.5 μm slices). For live cell imaging, micro-patterned substrates were mounted in a 35 mm circular dish using an UV curable glue (Norland Optical Adhesive 61). Keratinocytes were seeded as described above and imaged over 24 h using a Zeiss Axio Observer microscope (63 × 1.4NA, Oil, HCX Plan-Apo lens; XYZ motorised stage from Zeiss; temperature, moisture and CO2 were controlled using an environmental chamber from Zeiss and bubbling through water). Imaging was started 2 h after cell seeding, shortly after washing off non adhered cells with fresh medium. Images were acquired every 10 min using a CoolSnap HQ2 camera controlled by AxioVision v. 4.8.1 software and exported as a video at a rate of 10 frame/s.

### 2.5 Data and statistical analysis

Generation and analysis of heatmaps. Clusters (100 μm) formed of 5–10 cells (determined by the number of nuclei visible in the DAPI channel) were analysed. For smaller (50 μm) and larger (200 μm) clusters, the cell density per island was scaled proportionally to the change in island area (2–3 and 20–40 cells, respectively). Single cluster images were acquired (63 × 1.25NA, Oil lens) using the F-Actin channel for focussing (images were focused on the peripheral microfilament features for all images), alignment and cropping (using ImageJ software, see Fig. 1d). The resulting collection of images was stacked prior to splitting colour channels and overlaying each channel independently (Z Project/Sum Slices in ImageJ) into a greyscale image. The corresponding heatmaps were obtained using Origin (pixels with intensities below 30% of the maximum pixel intensity were set as black background). For quantification of involucrin and nuclear areas, greyscale overlays of 20–40 single cluster images were generated for each biological replicate (at least 3 replicates). The resulting image was thresholded (ImageJ), setting the minimum value to 30% of the maximum pixel intensity (after correction with the background level) and a particle size analysis was carried out to determine the area of 30% involucrin or DAPI intensity. When less than 20 cells were used to generate overlays and heatmaps, artefacts started to dominate (the average involucrin area decreased to that typical of single cells and the standard error increased; Supplementary Fig. 3).

Determination of % involucrin positive clusters and cell numbers per cluster. Cluster array images were acquired using a 10 × 0.3 NA lens. The involucrin and DAPI intensities of each single cluster (no constraint on cell numbers were applied at this stage) were measured (ImageJ, integrated density measurement) and corrected for background levels. In order to correlate DAPI intensities to cell numbers, for each experiment and condition, the DAPI intensities associated to 2, 4 and 6 nuclei exactly were measured (20 points per cell number). A linear fit allowed extrapolation of cell numbers from DAPI intensities of each clusters. The average number of nuclei were determined by extrapolation from the average DAPI intensity (no constraint on cell number was used). The % of involucrin positive clusters was determined by selecting and sorting clusters with 5–10 cells (extrapolated from DAPI intensities) and measuring the number of clusters above background level (determined as 3 × the average involucrin intensity of clusters 2 h after seeding).

All data were analysed by Tukey's test for posthoc analysis. Significance was determined by *P < 0.1, **P < 0.05, ***P < 0.01. A full summary of statistical analysis is provided in a separate .xlsx file (Stat.xlsx).

## 3 Results

### 3.1 Formation of micro-epidermis

Since the maximum diameter of a circular ECM coated island that can be covered by a single epidermal cell is approximately 50 μm [8,12], we determined that 100 μm diameter islands represented the smallest size on which to reproducibly build a multi-layered epidermis (Supplementary Fig. 1). To encourage the localization of a compartmentalised micro-epidermis, we designed a series of collagen I coated rings of 100 μm diameter presenting central non-adhesive disks of varying diameters (0–60 μm, Fig. 1). The non-adhesive regions are defined by micro-patterned polymer brushes to control cell spreading as described previously [7]. In contrast to single cell islands [8,13], keratinocytes spreading on collagen rings formed cell–cell contacts and experienced subtle differences in cell–cell and cell–ECM adhesions depending on their position in the assembly (periphery or centre) and the size of the non-adhesive patch (Fig. 1). We seeded disaggregated human epidermal cells onto the micro-patterned substrates and allowed the cells to attach for 1 h before washing off non-adherent cells. As previously described, undifferentiated keratinocytes expressing stem cell markers such as integrins adhered to the substrates, whereas differentiated cells did not. By 24 h some of the cells had initiated terminal differentiation and expressed involucrin and transglutaminase 1. The differentiated cells were not attached to the substrates, but had stratified and were attached to basal layer cells, thereby recapitulating the organisation of basal and differentiated cells in the original tissue (Fig. 1a, b). In the most densely populated clusters, differentiated cells sometimes expressed involucrin at the cell periphery rather than homogenously throughout the cytoplasm (Supplementary Fig. 2). Several hundreds of micro-epidermises containing 5–10 cells were generated on each micro-patterned substrate and exhibited highly reproducible cellular organisation (Fig. 1c).

To quantify the distribution of cells, images of individual micro-tissues (typically 20 to 40 for each biological replicate, Online Methods and Supplementary Fig. 3) were aligned and overlaid. In this way we generated heatmaps conveying precise information regarding the spatial distribution of particular molecules, such as involucrin, DNA, and keratins (Fig. 1d). In addition, we measured the area delimiting 30% of maximal fluorescence intensity as a measure of the localisation of a particular marker within each micro-epidermis (Fig. 1d–f). Involucrin occupied a significantly smaller area on 100 μm solid substrates (disks) than keratins, reflecting the fact that involucrin was expressed by differentiating, suprabasal cells in the centre of the substrates, whereas all cells on each island expressed keratins (Fig. 1e, f). For collagen I-coated disks without a cell resistant centre, the involucrin and nuclear areas were measured to be 3000 ± 200 and 2300 ± 100 μm^2^, respectively. When the size of the disk was varied (50–200 μm), the ratio of involucrin area to total island area was found to be lower for 100 μm disks (Supplementary Fig. 1). Hence 100 μm disks were used for further studies.

Cells cultured on islands with a non-adhesive centre (rings) of up to 40 μm initially (first 2 h) remained at the periphery of the pattern, spreading solely on the matrix-coated component. However, by 4–6 h intercellular contacts extended across the non-adhesive centre and the distribution of cell nuclei changed from the periphery to the centre of each island (Fig. 1e and Supplementary Video 1). In the case of islands with non-adhesive centres of 50 and 60 μm, cells did not cover the island centre completely, and as a result, involucrin positive cells did not partition as tightly to the centre of each island (Fig. 1e, f). Interestingly, involucrin positive cells on islands with a 40 μm non-adhesive centre (rings) partitioned more tightly to the centre (area of 2400 ± 100 μm^2^; Fig. 1f) than cells on islands that were uniformly adhesive (disks) (3000 ± 200 μm^2^).

The following is the Supplementary data related to this article:

Video S1 Spreading of cells on ring.

**Fig. 2:**
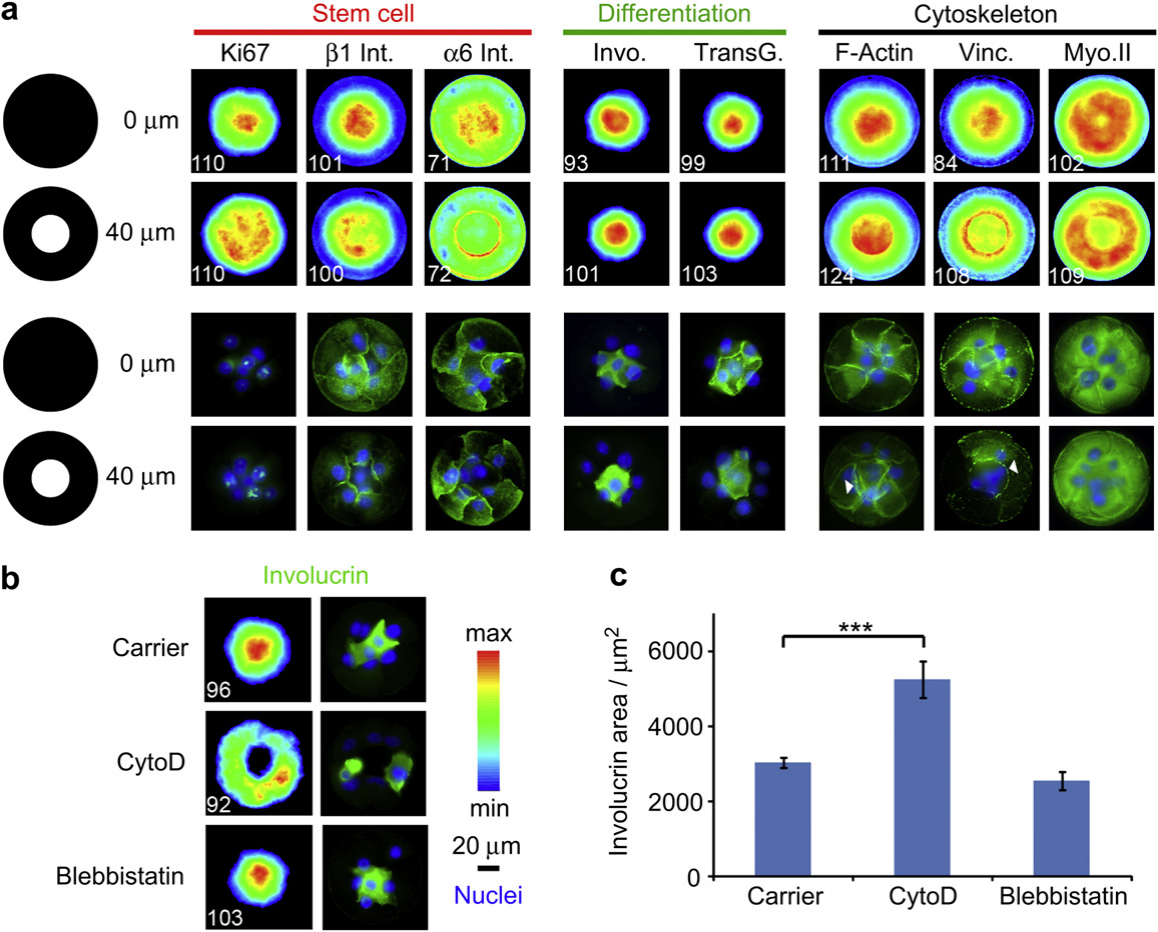
Engineering of micro-epidermis compartmentalisation and role of the cytoskeleton. (a) Heatmaps (top two rows) and single cluster (bottom two rows) images of stem cell, differentiation or cytoskeleton markers (green) and DAPI (blue). Clusters generated with collagen I coated disks (top) and rings (bottom, 40 μm non-adhesive patch) are compared. White numbers (bottom left of each heatmap) are the number of images overlayed. (b) Heatmaps (left) and single cluster images (right column; involucrin, green; DAPI, blue) of cells treated with carrier (DMSO), cytochalasin D (cytoD, 1 μM) and blebbistatin (3 μM). (c) Involucrin areas extracted from heatmaps. ***P < 0.01; error bars are s.e.m., *n* = 3(For interpretation of the references to colour in this figure legend, the reader is referred to the web version of this article.).

To validate the conclusion that cells could assemble a micro-epidermis on 100 μm diameter disks and rings, we examined additional markers of the basal and suprabasal interfollicular epidermal cell layers (Fig. 2). Cells expressing the differentiation marker transglutaminase 1 showed a similar distribution to cells expressing involucrin, partitioning more tightly on rings with a 40 μm non-adhesive centre than on uniformly adhesive disks (Fig. 2a). We observed that total cell nuclei had a central location on both collagen disks and rings; however, this was not the case for proliferative basal cell nuclei (Ki67 positive) (Fig. 2a). A similar effect of island type was observed on the heatmaps of the basal layer marker β1 integrin, although not as marked. However, α6 integrins showed a clearer concentration at the periphery of the non-adhesive centres (Fig. 2a). This is likely to reflect the different types of adhesive structures formed by these integrins: β1 integrins localise in focal adhesions, while α6β4 is a component of hemidesmosomes. The lack of matrix adhesion in the centre of the pattern excludes undifferentiated cells from this portion of space and favours the segregation of differentiated cells, based on their up-regulation of cell–cell adhesive molecules (E-cadherin, desmoglein and desmocollin isoforms 1 and 3) [14–16]. The change in the balance of cell-matrix and cell–cell adhesive interactions leads to better organization of cells into a micro-epidermis on collagen rings as compared to disks.

### 3.2 Role of cytoskeleton integrity and cell contractility in micro-epidermis assembly

To explore the mechanisms underlying micro-epidermal organisation we first examined the actin cytoskeleton. We found that keratinocytes on uniformly adhesive collagen-coated disks assembled actin fibres at the periphery of the epidermis and at cell–cell junctions (Fig. 2a). The actin at cell–cell borders converged in the centre of the disk, mainly within a 30 μm radius, as reflected in the maximum intensity of the resulting actin heatmap. On collagen-coated rings, the distribution of actin was similar, except that stress fibres concentrated at the periphery of the non-adhesive centre, reminiscent of stress fibres generated by single cells spreading over non-fouling components [7,13] (Fig. 2a) and resembling the actin ‘purse string’ that forms during closure of embryonic wounds [17]. Vinculin images and heatmaps showed marked intensity maxima at the centre of disks but not rings; in the latter case, vinculin was concentrated at the periphery of the non-adhesive area (Fig. 2a). Finally, myosin IIa heatmaps clearly showed peripheral maxima, especially in the case of rings, suggesting that cells in the centre of clusters were less contractile (Fig. 2a).

To examine the role of the actin cytoskeleton in the micro-epidermis assembly, 1 μM cytochalasin D was added 1 h after plating cells on rings with a 40 μm diameter non-adhesive centre. Cytochalasin D treatment inhibited actin polymerisation and prevented keratinocytes from closing over the centre of the islands. Cells still underwent terminal differentiation [8] but now involucrin positive cells remained at the periphery of the island (Fig. 2b). As a result the involucrin positive areas were greater in Cytochalasin D treated than in control islands (Fig. 2c).

In contrast to the effect of Cytochalasin D, cells seeded on islands and treated with blebbistatin, a myosin II inhibitor that inhibited stress fibre formation, did not display any aberrant architecture (Fig. 2b, c). Although blebbistatin did not prevent micro-epidermis formation it did interfere with cell motility. Live DIC imaging showed that, in the absence of the drug, cells plated on rings initiated a concerted circular motion a few hours after covering the non-adherent centre (Supplementary Video 1), a behaviour observed in other cell types [18]. Blebbistatin treatment decreased the speed at which ring closure occurred and inhibited collective cell motility, although single cells were motile and exchanged position with neighbours (Supplementary Video 2). We conclude that complete perturbation of the F-actin cytoskeleton resulted in total disaggregation of clusters, whereas abrogation of myosin-generated contractility had no effect on micro-epidermal architecture.

The following is the Supplementary data related to this article:

Video S2 Concerted circular motion of blebbistatin treated cells.

### 3.3 Functional redundancy of adherens and desmosomal junctions in micro-epidermis assembly

**Fig. 3:**
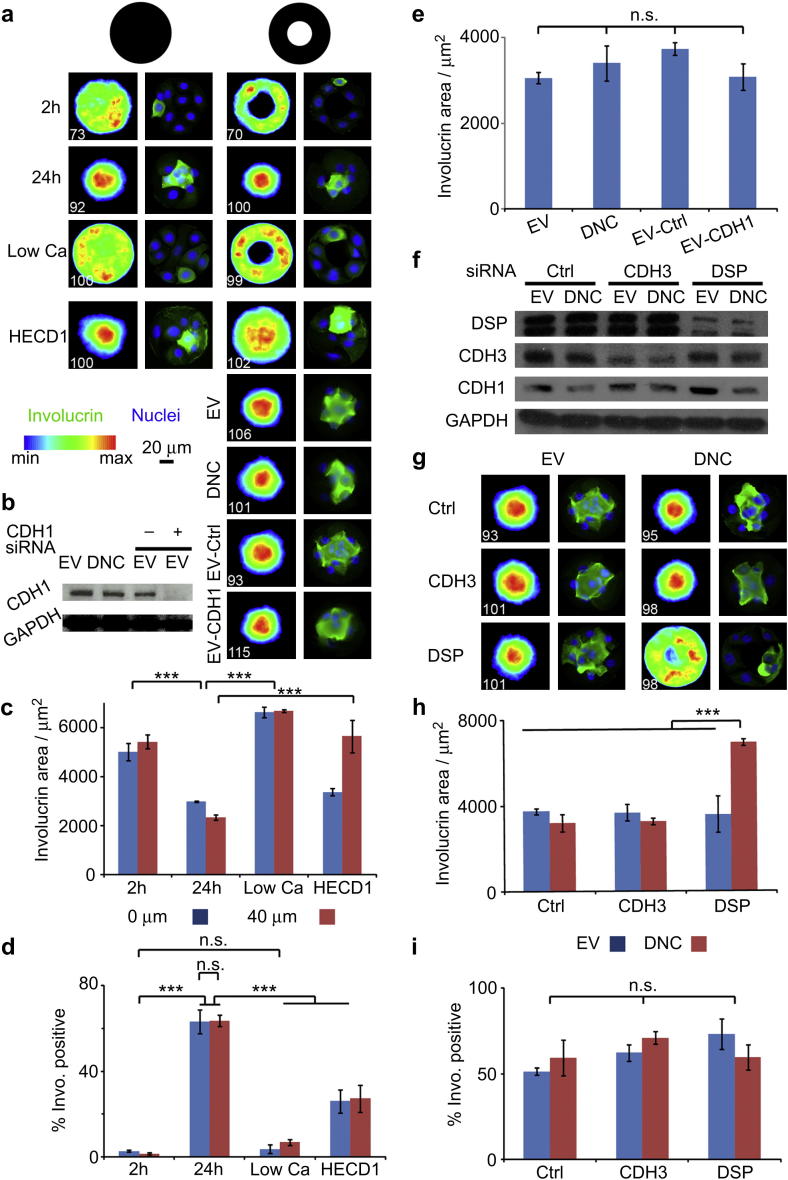
Micro-epidermis architecture is determined by redundant cadherin-mediated interactions. (a) Keratinocytes were cultured on collagen discs and rings in high calcium medium for 2 and 24h, low calcium (Low Ca, 24 h), and high calcium with an E-cadherin function blocking antibody (HECD1, 0.5 μg/mL). Empty vector (EV), dominant negative E-cadherin (DNC) expressing keratinocytes and EV keratinocytes treated with non-silencing (EV-Ctrl) and E-cadherin (EV-CDH1) siRNAs were incubated on collagen rings for 24 h. Involucrin heatmaps (left columns) and single cluster images (right columns; DAPI, blue; involucrin, green). White numbers (bottom left of each heatmap) are the number of images overlayed. (b) Western blot of EV, DNC, EV-Ctrl and EV-CDH1 cell lysates showing E-cadherin knock down and loading control (GAPDH). (c) Involucrin areas extracted from corresponding heatmaps for keratinocytes cultured in different media conditions. (d) Corresponding % of involucrin positive clusters (e) Involucrin areas extracted from heatmaps for EV, DNC, EV-Ctrl and EV-CDH1 keratinocytes. (f) EV and DNC keratinocytes were treated with non-silencing (Ctrl), P-cadherin (CDH3) and desmoplakin (DSP) siRNAs. Western blot of respective cell lysates showing the extent of protein knock down and loading control (GAPDH). (g) Corresponding heatmaps (left columns) and single cluster (right columns) images (involucrin, green; DAPI, blue). (h) Involucrin areas extracted from involucrin heatmaps after 24 h incubation on collagen rings (40 μm non-adhesive patch). (i) Corresponding % of involucrin positive clusters. ***P < 0.01; n.s. non significant, *P* > 0.1; error bars are s.e.m., *n* ≥ 3(For interpretation of the references to colour in this figure legend, the reader is referred to the web version of this article.).

Epidermal stratification is dependent on formation of adherens junctions and desmosomes, both of which are calcium-dependent intercellular adhesive junctions. When cells are cultured in low calcium medium they cannot stratify and cells that undergo terminal differentiation detach from the substrate into the culture medium. We confirmed that this was also the case when cells were seeded on 100 μm diameter disks or on rings with a 40 μm diameter non-adhesive centre. Low calcium culture conditions completely disrupted cell–cell junctions and led to total disorganisation of cell clusters (Fig. 3a, c). The number of cells per island was unaffected (results not shown) but the proportion of islands that contained involucrin positive cells was greatly reduced (Fig. 3d).

E-cadherin is the major component of epidermal adherens junctions. Treatment with a function blocking antibody (HECD1) to E-cadherin partially perturbed segregation of involucrin positive cells into the centre of islands, the effect being stronger on rings than on disks (Fig. 3a, c). However, the disruption was less pronounced than when cells were plated in low calcium medium and heatmaps showed that the maximum involucrin intensity was still localised in the centre of each cell cluster. In addition, while the number of cells per disk was unaffected by HECD1 (results not shown), there were significantly less involucrin positive clusters (Fig. 3d). In contrast, siRNA-mediated depletion of E-cadherin (CDH1) had no effect on partitioning and differentiation (Fig. 3a, b and e).

To further test the role of E-cadherin mediated adhesion in keratinocyte differentiation and cluster self-segregation, we infected primary human keratinocytes with a dominant negative E-cadherin chimera (DNC) in which the extracellular domain of E-cadherin is replaced with that of H-2K^d^ [11]. In conventional cultures DNC efficiently displaces endogenous E-cadherin from cell–cell junctions and inhibits stratification. However, cells expressing DNC did not show any significant increase in involucrin area compared to those expressing an empty vector (involucrin area: EV, 3100 ± 100 μm^2^; DNC, 3400 ± 400 μm^2^; Fig. 3a, e). Immnuno-fluorescence confirmed that E-cadherin was displaced from the cell–cell junction, but not other cell–cell adhesion markers (P-cadherin and desmoplakin, Supplementary Fig. 4). The number of involucrin positive clusters and the average cell density per pattern remained unchanged (results not shown). Thus the effect of DNC also contrasted with that of the HECD1 antibody.

To explore further the roles of adherens junctions and desmosomes in micro-epidermis assembly we knocked down the expression of P-cadherin (CDH3) and the major plaque component, desmoplakin (DSP, Fig. 3f). In vivo, conditional ablation of E-cadherin combined to knock down of P-cadherin resulted in blistering of the epidermis and impaired its barrier function [19], whereas desmoplakin knock down alone significantly perturbed epidermal sheet formation and mechanical integrity [20]. Our combined knock down approach showed that P-cadherin knock down did not have a significant impact on cluster assembly (Fig. 3f–h). Desmoplakin knock down alone did not result in impairment of cell segregation (involucrin area of EV with DSP siRNA, 3600 ± 900 μm^2^ compared to EV with control siRNA, 3700 ± 100 μm^2^) (Fig. 3g, h) and did not affect the number of cells per island (results not shown) or proportion of islands with involucrin positive cells (Fig. 3i). E-cadherin and P-cadherin remained at cell–cell junctions although DSP was displaced (Supplementary Fig. 4). However, when expression of DSP siRNA was combined with expression of the dominant negative E-cadherin mutant (DNC), complete disruption of cluster architecture (involucrin area, 6900 ± 200 μm^2^) was observed, phenocopying cytochalasin D-treated clusters (Fig. 3g, h). E-cadherin and P-cadherin were also displaced from cell–cell junctions (Supplementary Fig. 4). The disruption of cluster assembly was not accompanied by any change in the proportion of involucrin positive islands (Fig. 3i) or the number of cells per island (results not shown). We conclude that adherens and desmosome junctions exhibit functional redundancy in determining the architecture of micro-epidermis.

### 3.4 Perturbed stratification of tumour cells

**Fig. 4:**
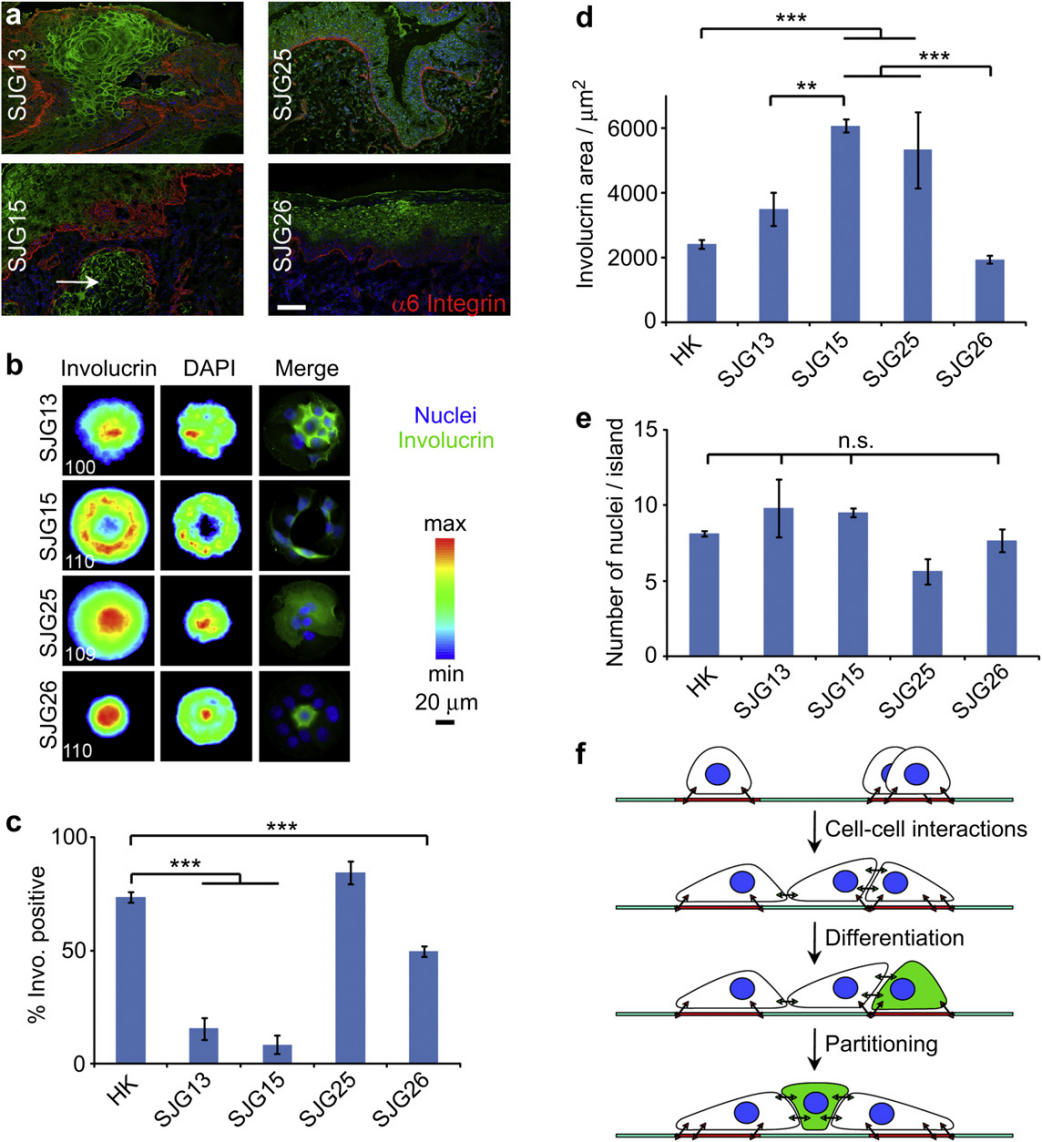
Disrupted architecture of cancerous micro-epidermis. (a) Tumour skin sections immuno-stained for involucrin (green), α6 integrin (red) and DAPI (blue; scale bar, 200 μm). The white arrow points towards an island of keratinocytes invading into the underlying dermal layer. (b) Cancer cells were allowed to adhere to collagen rings (40 μm non-adhesive patch) for 24 h before fixation and immuno-staining. Heatmaps (left and middle columns) and single cluster images (right column; involucrin, green; DAPI, blue). White numbers (bottom left of heatmaps) are the number of images overlayed. (c) Corresponding % of involucrin positive clusters. (d) Involucrin areas extracted from the respective heatmaps. (d) Corresponding cell densities. (f) Side-view scheme representing the adhesion and spreading of cells on collagen rings and their formation of compartmentalised micro-tissues. **P < 0.05; ***P < 0.0; n.s., non significant, *P* > 0.1; error bars are s.e.m.; *n* = 3(For interpretation of the references to colour in this figure legend, the reader is referred to the web version of this article.).

Squamous cell carcinomas (SCCs) are tumours of multi-layered epithelia, such as the epidermis and lining of the mouth. Hallmarks of SCC are increased proliferation, reduced differentiation and disturbed tissue architecture [21]. To examine whether the behaviour of SCC cells on micro-patterned rings correlates with their in vivo behaviour, we examined cells from four independent oral cavity tumours, for which biopsies of the original tumours were available (Fig. 4a). The number of cells that attached per island did not differ significantly between lines except for a slight decrease in the case of SJG25 (Fig. 4e).

One of the cell lines, SJG26, formed a micro-epidermis with a similar architecture to normal epidermal cells (Fig. 4b). SJG26 cells plated on rings with a 40 μm non-adhesive centre were able to bridge over the centre. The proportion of islands with involucrin positive cells was lower than in the case of normal keratinocytes (Fig. 4c) but the involucrin positive cells partitioned in the centre of the islands to a similar extent to normal keratinocytes (Fig. 4d). The relatively normal behaviour of SJG26 cells correlated well with the histology of the lesion from which they were derived, which was characterised by preservation of basal and suprabasal compartments (Fig. 4a).

The remaining three tumour cells (SJG13, SJG15 and SJG25) exhibited abnormal architecture on rings with a 40 μm non-adhesive centre (Fig. 4b, d). SJG25 cells generated the same proportion of involucrin-positive clusters as normal keratinocytes (Fig. 4c). However, the cells did not partition to the centre of the islands as effectively as normal cells (Fig. 4b, d). SJG13 and SJG15 cells exhibited a marked reduction in the proportion of involucrin-positive clusters (Fig. 4c). However, whereas SJG15 cells failed to form a stratified micro-epidermis, resulting in a lack of partitioning of involucrin positive cells, segregation of involucrin positive SJG13 cells was almost normal (Fig. 4b, d). Whereas E-cadherin, P-cadherin and DSP were completely displaced from cell–cell junctions in SJG15 cells, they exhibited a largely normal localisation in other cancer cells (Supplementary Fig. 5). SJG13, 15 and 25 tumours all exhibited disturbed tissue architecture (Fig. 4a), but SJG15 was the tumour that had the worst prognosis. On presentation the SJG15 tumour already had islands of keratinocytes invading into the underlying dermal layer (see arrows, Fig. 4a). We conclude that the behaviour of SCC cells on adhesive rings mimics the properties of the tumours from which they are derived.

## 4 Discussion

Although many useful insights into cell–microenvironmental interactions can be gained by studying those interactions at the single cell level, there are additional physical phenomena that govern tissue assembly and can only be studied in multi-cellular complexes. These include reaction-diffusion [22,23], differential interfacial tension [24], differential motility [25] and differential adhesion [26,27]. Perturbation of such cues can result in altered tissue architecture and physiology [3]. The method that we describe provides a tool to study quantitatively how cell-ECM and cell–cell adhesive interactions influence formation of a stratified epithelium, the epidermis, by stem cells and their progeny.

The steps in micro-epidermis formation are shown schematically in Fig. 4f. Generation of a micro-epidermis depends on differences in the adhesive properties of stem cells and differentiated cells. Stem cells adhere preferentially to the ECM [28]. During terminal differentiation, cells downregulate integrin expression, assemble increased numbers of adherens junctions and desmosomes, and change the protein composition of the junctions. As a result, the two subpopulations of cells sort out [14,29]: stem cells remain attached to the ECM while differentiating cells detach from the ECM and adhere to the apical surface of stem cells and to neighbouring suprabasal cells.

Cell–ECM and cell–cell adhesion both require assembly of the actin cytoskeleton. Although keratinocyte colonies expand via the combination of proliferation and migration of cells at the periphery of clones [30], we found that coordinated cell motility and myosin contractility [31,32] were not required to generate micro-tissues. This is somewhat surprising considering that myosin is reported to be necessary for the establishment and maintenance of adherens junctions [33–35], by enabling the transmission of tension through cell–cell adhesion complexes and reinforcement of the associated cortical actin cytoskeleton. The reason for the lack of an effect of blebbistatin may be that, in keratinocytes, myosin IIa does not localise at cell–cell junctions [31,33] (Fig. 2a). In contrast to the epidermis, blebbistatin and other myosin inhibitors perturb the partitioning of osteoblasts and mammary epithelial cells [27,36,37].

Assembly of micro-epidermal clusters was completely disrupted in low calcium, consistent with the failure of differentiating cells to form adherens junctions and desmosomes and to downregulate integrin expression in low calcium medium [14,38]. In standard calcium medium, combined inhibition of adherens junctions and desmosomal junctions was necessary to completely inhibit stratification (Fig. 3). The implication of desmosomes in the partitioning of micro-epidermis is consistent with the impaired epidermal sheet integrity observed in desmoplakin knock out mice [20] and the development of epidermal diseases associated with desmoplakin mutations or autoantibody detection [39,40]. Similarly, desmosomal adhesions have been implicated in alveolar morphogenesis of mammary epithelial cells [41]. In addition, although evidence for redundancy between adherens and desmosomal junctions has been reported in parental A431 epithelial cells [42], it had not been directly associated with human epidermal architecture and integrity. Finally, the altered cluster architecture did not correlate with changes in differentiation levels, in line with previous reports giving evidence that stratification was regulated separately from the initiation of terminal differentiation [14,43].

Altered cell–ECM and cell–cell interactions have been implicated in cancer [44,45]. Abnormal integrin expression plays a role in SCC formation and tumour progression [28]. Decreased E-cadherin expression and desmosome formation are correlated with poor prognosis in tumours [45,46]. In line with such concepts, SCC-derived cell lines displayed a reduced ability to stratify on adhesive islands that was distinct from their ability to differentiate. The behaviour of cells on the islands correlated well with the properties of the tumours from which they were derived. Thus SJG26 cells, derived from an area of hyperplastic mucosa surrounding an invasive lesion, resembled normal keratinocytes in their ability to form a micro-epidermis. SJG15 cells, derived from a tumour that showed early recurrence and high metastatic potential, had the most disrupted micro-epidermal phenotype and key junctional proteins (E-ad P-cadherin and DSP) were displaced from the cell–cell interface. These results suggest possible correlations between tissue morphology and gene expression in SCCs, as observed in breast cancer cell lines [47].

## 5 Conclusion

The method presented here for creating micro-patterned multi-cellular assemblies recapitulates some of the key architectural features of the interfollicular epidermis. It allows quantitative studies of the mechanisms that control normal tissue assembly and maintenance and how those mechanisms are disrupted in tumours. Since the tissue-like structures assembled on micro-patterned substrates is highly reproducible and requires low cell numbers, it lends itself to high throughput, automated screens of drugs that normalise tissue architecture.

## Appendix A Supplementary data

Impact of collagen island diameter on segregation, image of micro-epidermis displaying cell-peripheral involucrin staining, impact of number of clusters analysed to generate heatmaps, effect of DNC expression on cell–cell junction composition, composition of the intercellular junctions of normal keratinocytes, SCC cells and videos displaying the motile behaviour of cell clusters in control conditions and after blebbistatin treatment and statistical analysis results can be found at http://www.sciencedirect.com.
