# Supplementary figures and images for: Mimicking normal tissue architecture and perturbation in cancer with engineered micro-epidermis
Source: Biomaterials. 2012 Jul;33(21):5221–9. doi: 10.1016/j.biomaterials.2012.04.009 (PMC3437971; doi:10.1016/j.biomaterials.2012.04.009)

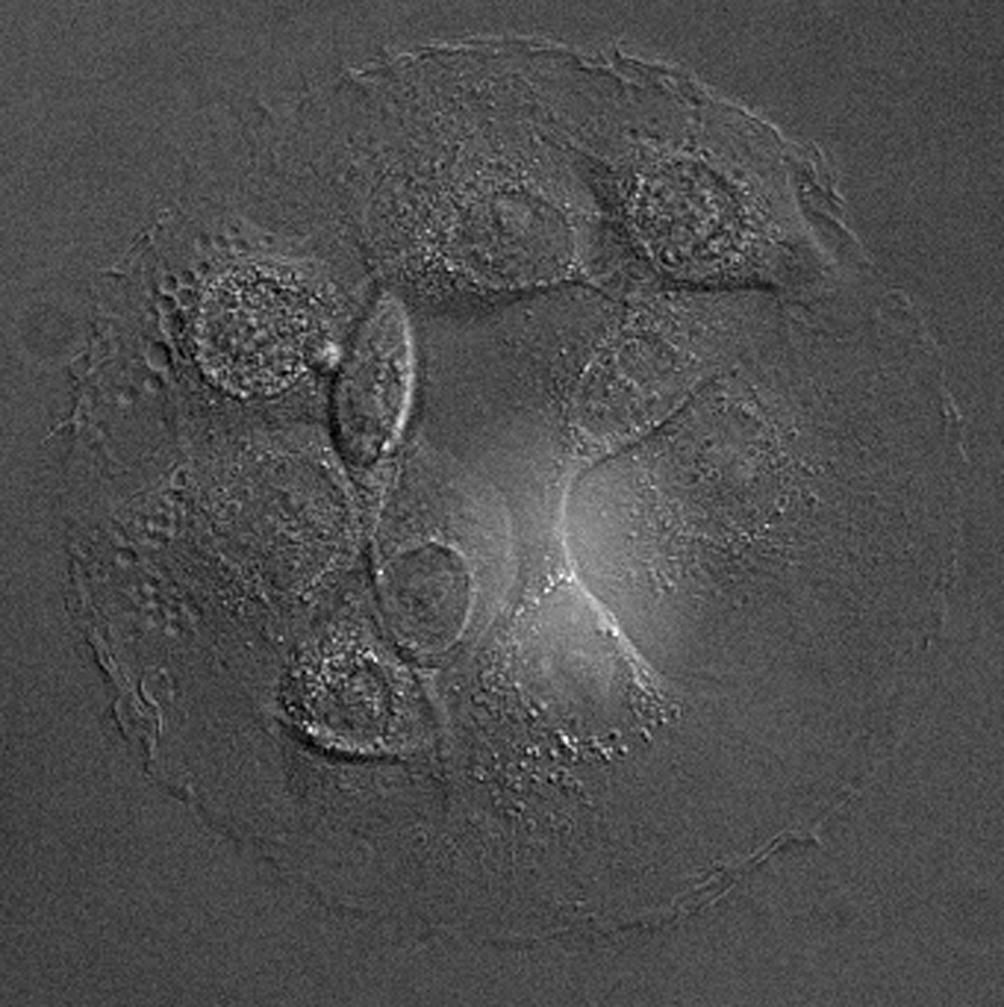

Supplement: Video S2 — Concerted circular motion of blebbistatin treated cells. [file mmc2.jpg]
