# Supplementary material for: Mimicking normal tissue architecture and perturbation in cancer with engineered micro-epidermis
Source: Biomaterials. 2012 Jul;33(21):5221–9. doi: 10.1016/j.biomaterials.2012.04.009 (PMC3437971; doi:10.1016/j.biomaterials.2012.04.009)
Supplement: Supplementary file 4 [file mmc4.pdf]

Mimicking Normal Tissue Architecture and Perturbation in Cancer with Engineered Micro-Epidermis

Julien E. Gautrot,[1,2,3] *Chunming Wang,*[1,2] *Xin Liu,*[1] *Stephen J. Goldie,*[4] *Britta Trappmann,*[1] *Wilhelm T. S. Huck*[1,5*] *and Fiona M. Watt*[2,4*]

[1] Melville Laboratory for Polymer Synthesis, Department of Chemistry, University of Cambridge, Lensfield Road, Cambridge, CB2 1EW, UK.

[2] Wellcome Trust Centre for Stem Cell Research, University of Cambridge, Tennis Court Road, Cambridge, CB2 1RE, UK.

[3] School of Engineering and Materials Science, Queen Mary, University of London, Mile End Road, London, E1 4NS, UK.

[4] Cancer Research UK Cambridge Research Institute, Li Ka Shing Centre, Robinson Way, Cambridge, CB2 0RE, UK.

[5] Radboud University Nijmegen, Institute for Molecules and Materials, Heyendaalseweg 135, 6525 AJ Nijmegen, The Netherlands.

* To whom correspondence should be addressed E-mail: fiona.watt@cancer.org.uk, w.huck@science.ru.nl.

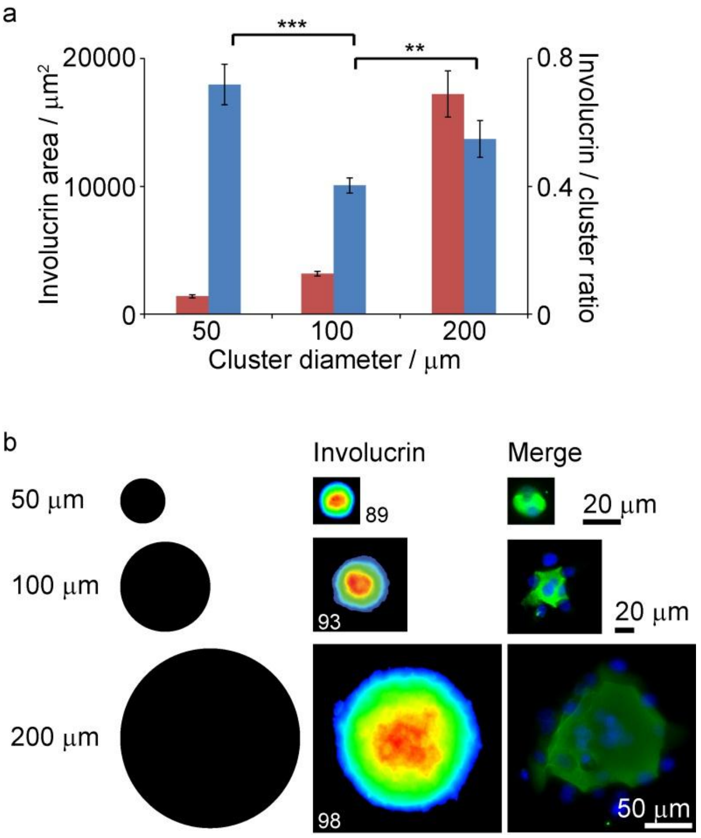

Supplementary Figure 1. Impact of collagen island diameter on cell segregation. Undifferentiated keratinocytes adhered to collagen I disks of varying size (50, 100 and 200 µm in diameter) for 24h before fixation and immuno-staining. (a) Involucrin areas (red) and ratios of involucrin areas to total cluster areas (blue) extracted from heatmaps. ** P < 0.05; *** P < 0.01; error bars are s.e.m., n ≥ 3. (b) Single collagen island size (left column), heatmaps (middle) and single cluster images (right; involucrin, green; DAPI, blue). Numbers at the bottom left of heatmaps (for 100 and 200 µm islands and on the right next to the heatmap of a 50 µm island) are the number of images overlayed. These results show that for islands of smaller diameters (50 µm), the ratios of involucrin area to total island area markedly increased. Larger adhesive islands (200 µm) displayed normal architecture, but ratios increased modestly compared to 100 µm islands.

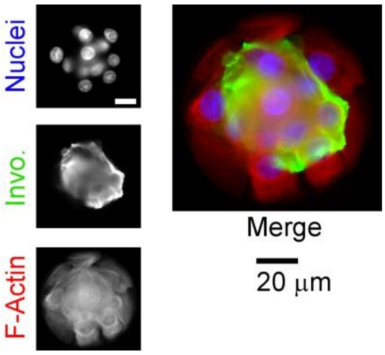

Supplementary Figure 2. Micro-epidermis displaying cell-peripheral involucrin staining. Undifferentiated keratinocytes were allowed to adhere to collagen discs for 24 h before fixation and staining for involucrin. Involucrin expressed by the differentiated cell in the centre of the cluster is confined to the cell periphery, a feature of the later stages of epidermal differentiation.

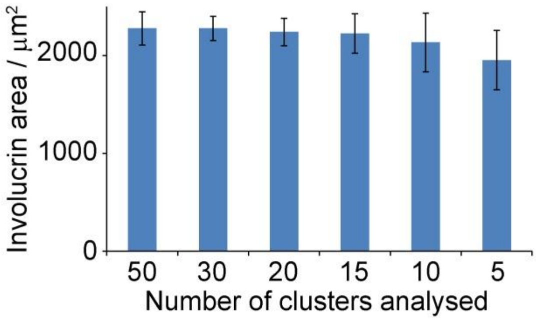

Supplementary Figure 3. Impact of the number of clusters analysed to generate heatmaps. Undifferentiated keratinocytes were allowed to adhere to collagen rings (40 μm non-adhesive patch) for 24 h before fixation and staining for involucrin. Images of the resulting clusters (100 in total) were randomly grouped in image sets composed of varying numbers (5-50; the "50" group contained only two sets). For each set of images, a heatmap was generated and the corresponding involucrin area measured, which was plotted against the number of images used for each image set. Error bars are s.e.m., $n \geq 3$ for $N = 5\text{-}30$ and $n = 2$ for $N = 50$.

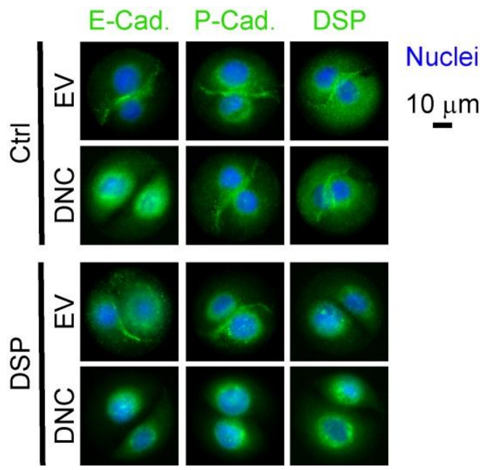

Supplementary Figure 4. Effect on cell-cell junction composition of expressing dominant negative E-cadherin (DNC) and DSP siRNA. EV and DNC keratinocytes were treated with non-silencing (Ctrl) and desmoplakin (DSP) siRNAs. Cells were cultured on 50 μm collagen discs in FAD for 24h before fixation and staining for E- and P-cadherin as well as DSP. Only islands displaying 2 cells are shown so that cells present comparable interfaces.

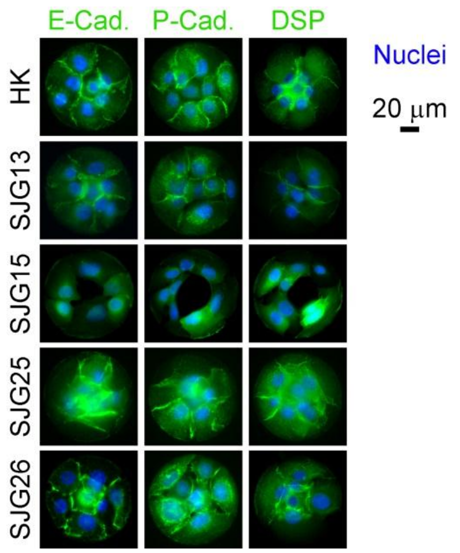

Supplementary Figure 5. Composition of the intercellular junctions of normal keratinocytes and SCC cells. Cancer cells were allowed to adhere to collagen rings (40 μm non-adhesive patch) for 24h before fixation and immuno-staining. Representative single cluster images (E-cadherin, P-cadherin and DSP, green; DAPI, blue).

Supplementary Video 1. Undifferentiated HEKs were seeded on a collagen I ring (40 µm non adhesive patch) and imaged using live DIC microscopy in complete FAD medium. Acquisition started 2h after seeding, every 10 min. Image width: 105 µm. 10 frames/s.

Supplementary Video 2. Undifferentiated HEKs were seeded on a collagen I ring (40 µm non adhesive patch) and imaged using live DIC microscopy in complete FAD medium containing blebbistatin (3 µM). Acquisition started 2h after seeding, every 10 min. Blebbistatin was added just before starting imaging. Image width: 105 µm. 10 frames/s.
